# AKF-PD alleviates diabetic nephropathy via blocking the RAGE/AGEs/NOX and PKC/NOX Pathways

**DOI:** 10.1038/s41598-018-36344-w

**Published:** 2019-03-13

**Authors:** Jiao Qin, Zhangzhe Peng, QiongJing Yuan, Qian Li, Yu Peng, Rui Wen, Zhaolan Hu, Jun Liu, Xiongfang Xia, Hong Deng, Xuan Xiong, Jinyue Hu, Lijian Tao

**Affiliations:** 1 Nephropathy Department, Changsha Central Hospital, South Shaoshan Road 161, Changsha, Hunan 410004 China; 2 Department of Anatomy and Neurobiology, School of Basic Medical Science, Central South University, Tongzipo Road 172, Changsha, Hunan 410008 China; 3 Department of Nephrology, xiang ya hospital, Central South University, 87 Xiangya Road, Changsha, Hunan 410008 China; 4 Medical Research Center, Changsha Central Hospital, South Shaoshan Road 161, Changsha, Hunan 410004 China

## Abstract

Diabetic nephropathy (DN) is a major complication of diabetes. Currently, drugs are not available to effectively control the disease. Fluorofenidone (AKF-PD) is a recently developed drug; it possesses activities in reducing DN progression in preclinical research. Nonetheless, its renal protection and the underlying mechanisms have not been thoroughly investigated. We report here that AKF-PD significantly alleviatesrenal oxidative stress (OS) in *db*/*db*mice through downregulation of Nicotinamide Adenine Dinucleotide Phosphate (NADPH) oxidase and upregulation of glutathione peroxidase and superoxide dismutase, thereby protecting kidney from DN pathogenesis. AKF-PD likely reduces OS through the advanced glycation end products (AGE) and protein kinase C (PKC) pathways. While renal AGEs, PKCα, PKCβ, and NADPH oxidase 4 (NOX4) were all substantially upregulated in *db*/*db* mice compared to *db*/*m* animals, AKF-PD robustly downregulated all these events to the basal levelsdetected in *db*/*m* mice. In primary human renal mesangial cells (HMCs), high glucose (HG) elevated receptor for advanced glycation endproducts (RAGE), PKCα, PKCβ and NOX4 activity, and induced the production of reactive oxygen species (ROS); these events were all inhibited by AKF-PD. Furthermore, HG led to mitochondrial damagein HMCs;AKF-PD conferred protection on the damage. Knockdown of either PKCα or PKCβ reduced HG-induced ROS production and mitochondrial damage in HMCs. The knockdown significantly enhanced AKF-PD-mediated inhibition of ROS production and mitochondrial damage in HG-treated HMCs. Collectively, our study demonstrates that AKF-PD protects renal function under diabetes conditions in part through inhibition of OS during DN pathogenesis. AKF-PD can be explored for clinical applications in DN therapy.

## Introduction

Diabetes mellitus (DM) is an endocrine metabolic disease that seriously affects human health. The incidence of DM has been rapidly rising in recent decades worldwide^1^. Diabetic nephropathy (DN) as the most common and serious complication of DM occurs in 20–40% patients with DM^2,3^. In addition, DN is the main cause for chronic renal failure and the primary cause of death for DM^2,3^. Unfortunately, there is no available drug that can effectively treat DN. Thus, it is of vital importance to clarify the mechanisms of DN and develop effective therapies.

The pathogenesis of DN is associated with OS, renal hemodynamic dysfunction, micro-inflammatory reaction, metabolic disorders, the production of multiple cytokines and vasoactive molecules like angiotensin II and endothelin, and mesangial cell proliferation as well as ECM accumulation^4–8^. Currently, the first-line therapy for chronic kidney disease involves angiotensin-converting enzyme inhibitor (ACEI) and Angiotensin II Receptor Blockers (ARB)^9–11^. Previous studies have reported that ACEI/ARB can protect renal function by primarily regulating glomerular hemodynamics, leading to reductions in urine protein and inhibition of renal fibrosis12. The limitation of the first-line therapy in targeting an avenue of multiple abnormalities likely explains the common elevation of SCR in the long-term users of ACEI/ARB. The elevation resulted in patients at risk of developing renal dysfunction^12^. Plenty preclinical experiments have been conducted to examine the anti-fibrosis pharmacology of numerous drugs, including statins, rhubard and tripterygium, poricoic acid, ergone and so on^4–7,13–17^. These drugs display activities in regulating cytokines secretion and reducing inflammatory reactions; their safety and clinical efficacy are under investigation.

It is emerging from a series of recent studies that OS is a critical cause of DN^8,9^. In mouse models for DN and patients, ROS and the metabolic product of peroxidation such as malondialdehyde (MDA) and 8-iso-PGF2a were elevated concurrently with decreases in antioxidase activity including SOD and GSH-Px, supporting the importanceof ROS during the course of DN. At early stages, ROS impairs the vascular permeability and glomerular hemodynamics, destroys the glomerular electrostatic and filtration barrier, activates nuclear factor kappa beta (NF-κB) and activator protein 1(AP-1), and regulates the secretion of multiple inflammatory mediators^10^. At advanced stages of DN, ROS can promote the transdifferentiation of renal tubular cells, induce apoptosis in renal podocytes and mesangial cells, alter the balance of ECM production and degradation, and accelerate the formation of glomerular sclerosis^11^. ROS thus promotes DN initiation and progression by affecting multiple DN processes. These properties indicate targeting ROS being attractive in DN therapy. However, this potential has yet to be realized owing to the lack of medications.

The basic pathological changes of DN is renal fibrosis caused by the accumulation of extracellular matrix (ECM), mainly including collagen I and collagen IV, fibronectin and vementin^18,19^. The reversal and prevention of renal fibrosis is the key to DN therapy. accumulated studies have confirmed that renal fibrosis is associated with oxidative stress (OS) and inflammation, which is related to tissue injury resulted in imbalance between the production and elimination of reactive oxygen species (ROS)^20–25^. Therefore, how to reduce ROS formation is of clinical significance to preclude DN of diabetic patients. Our research group has recently invented a very promising novel drug AKF-PD, also known as Fluorofenidone; AKF-PD delivers satisfactory results in the treatment of renal interstitial fibrosis in preclinical research^26,27^. AKF-PD dramatically delayed the progression of DN in *db*/*db* mouse, indicating its attractive potential in management of patients with renal fibrosis. But less is known about the therapeutic mechanism of AKF-PD in DN. In view of the important role of OS in the pathogenesis of DN, our previous research suggested that AKF-PD likely inhibited the progression of DN through suppression of the expression of NADPH oxidase^27,28^. It is known that the advanced glycation end products (AGEs) and the protein kinase C (PKC) signal pathway play a central role in the expression of NADPH oxidase/OS^29^, which might be the targets of AKF-PD in DN treatment. In this study, we aimed to detect the therapeutic effects of AKF-PD in DN and to explore the related molecular mechanism both *in vitro* and *in vivo*.

## Results

### AKF-PD reduces mesangial expansion of DN

**Figure 1:**
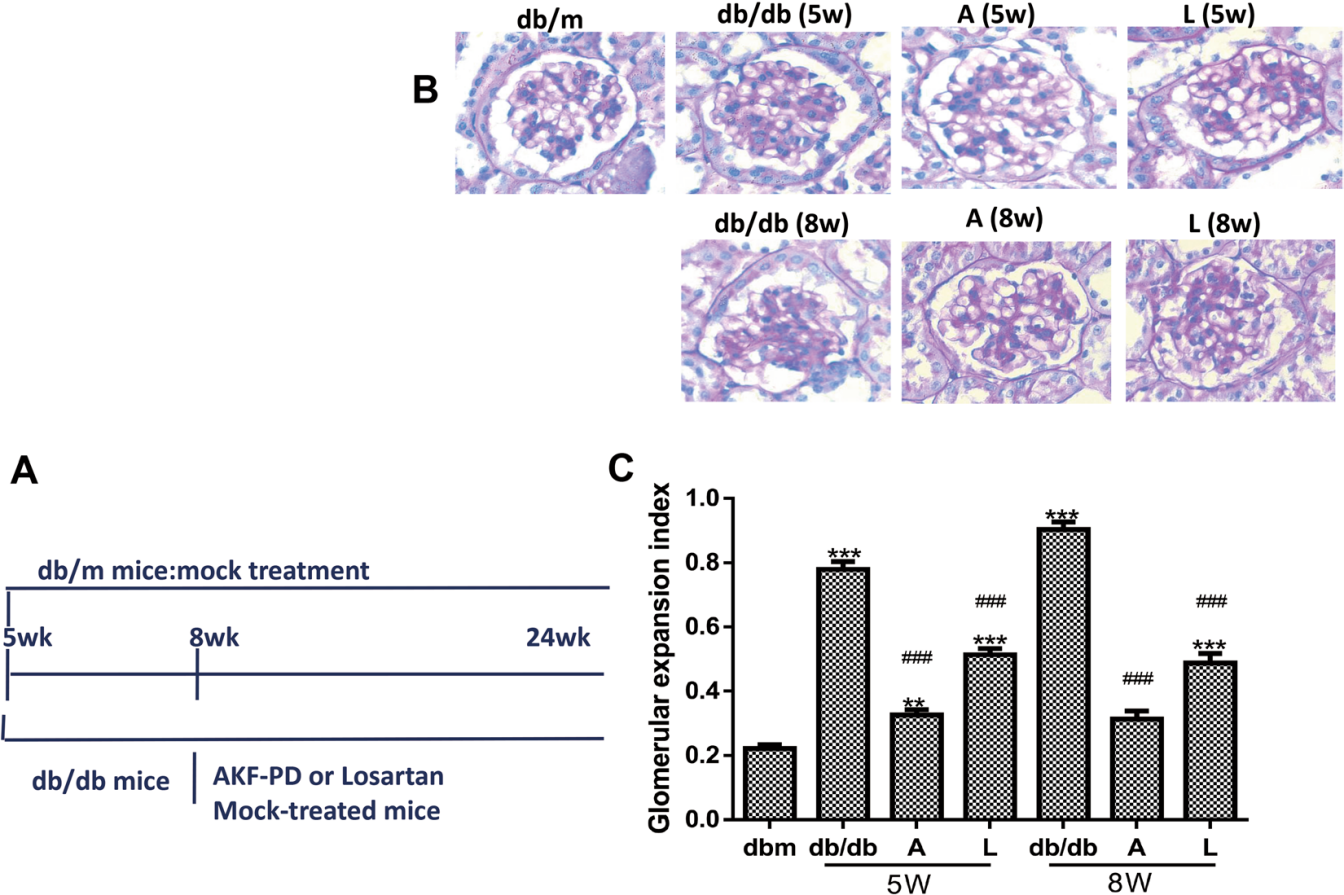
AKF-PD reduces glomerular ECM expansion in *db*/*db* mice. (**A**) Typical PAS staining of glomerulus of *db*/*m* mice and *db*/*db* mice receiving mock treatment, AKF-PD (**A**) or Losartan (L) staring at either 5 weeks (5w) or 8 weeks (8w) old till the end of experimental duration at 24 weeks. (**B**) Quantification of glomerular ECM expansion for the indicated genotypes and treatments (n = 6 for each group). ***p < 0.0001 in comparison to *db*/*m* mice; ^###^p < 0.0001 in comparison to *db*/*db* mice.

In comparison to *db*/*m* mice, renal mesangial matrix expansion was clearly observed in *db*/*db* mice (Fig. 1B). The addition of AKF-PD or losartan at week 5 (5w) and 8w significantly alleviated this expansion (Fig. 1B), which was evidenced by lowering renal glomerular expansion index (Fig. 1C). Therapeutic effect of the intervention initiated at week 8 seems better than weeks 5, evident by the non-significant glomerular expansion index observed in the treated *db*/*db* mice compared to *db*/*m* mice (Fig. 1C).

### AKF-PD reduces Albumin/creatine ratio

To demonstrate the above renal protection delivered by AKF-PD being functional, we examined whether renal function was also preserved. As expected, the renal function of *db*/*db* mice was significantly compromised compared with *db*/*m* mice (Table 1). Administration of AKF-PD lowered 24-hour urine albumin/creatinine ratio (ACR) (Table 1), regardless whether intervention was initiated at weeks 5 or 8-. This provides the first demonstration that kidney function can be partially preserved by AKF-PD at least in *db*/*db* mice. On the other hand, the administration of losartan aggravated renal injury with a significant increase in serum creatinine SCR (Table 1). Collectively, evidence suggests that AKF-PD is better than losartan indeceasing urine protein at 8 weeks (p < 0.05), at the same time we confirmed that AKF-PD did not influence uric acid and serum serum creatinine in AKF-PD treated mice compared to db/db mice.

**Table 1:** AKF-PD and losartan could protect the progression of diabetic nephropathy in db/db mice.

	*db*/*m*	*db*/*db*					
		Ctrl-5w	AKF-PD-5w	Losartan-5w	Ctrl-8w	AKF-PD-8w	Losartan-8w
BUN (mmol/L)	9.3 ± 1.22	9.1 ± 0.89	9.25 ± 1.22	11.9 ± 1.5^**##^	11.5 ± 0.87^*^	10.45 ± 0.75	11.86 ± 1.52^**^
SCR (µmol/L)	37.13 ± 2.42	42.23 ± 2.65	35.82 ± 2.85	68.63 ± 15.62^##^	48.67 ± 4.27	41.22 ± 4.15	69.37 ± 13.12
BUA (mmol/L)	246.67 ± 65.87	264.9 ± 74.57	242.73 ± 21.88	286.8 ± 138.54	349.17 ± 63.93^*^	345.88 ± 40.04*	438.72 ± 47.06^***^
ACR (µg/mg)	54.96 ± 8.43	503.24 ± 156.63^***^	94.46 ± 5.86^###^	498.39 ± 142.96^***^	650.38 ± 108.01^***^	127.55 ± 44.24^###§§§^	501.56 ± 121.53^***#^
GLU (mmol/L)	7.43 ± 0.86	61.7 ± 4.16^***^	59.50 ± 3.53^***^	59.85 ± 1.93^***^	73.83 ± 6.47^***^	62.5 ± 4.74^***^	69.85 ± 5.87^***^
BW (g)	31.62 ± 1.29	39.83 ± 1.46^***^	39.23 ± 0.77^***^	39.32 ± 1.91^***^	40.82 ± 1.92^***^	39.85 ± 1.35^***^	39.2 ± 091^***^

VS db/m *p < 0.05, **p < 0.01, ***p < 0.000.

VS corresponding db/db Ctrl group ^#^p < 0.05, ^##^p < 0.01, ^###^p < 0.000.

VS corresponding Losartan group ^§^p < 0.05, db/m, ^§§^p < 0.01, db/m, ^§§§^p < 0.000 BUN (mmol/L) blood urea nitrogen; SCR (μmol/L): serum creatinine; BUA (μmol/L): blood uric acid.

ACR (μg/mg): albumin/creatinine ratio; GLU (mmol/L): blood glucose; BW(g): body weight.

### Protective effects of AKF-PD on renal function are associated with the inhibition of OS

**Figure 2:**
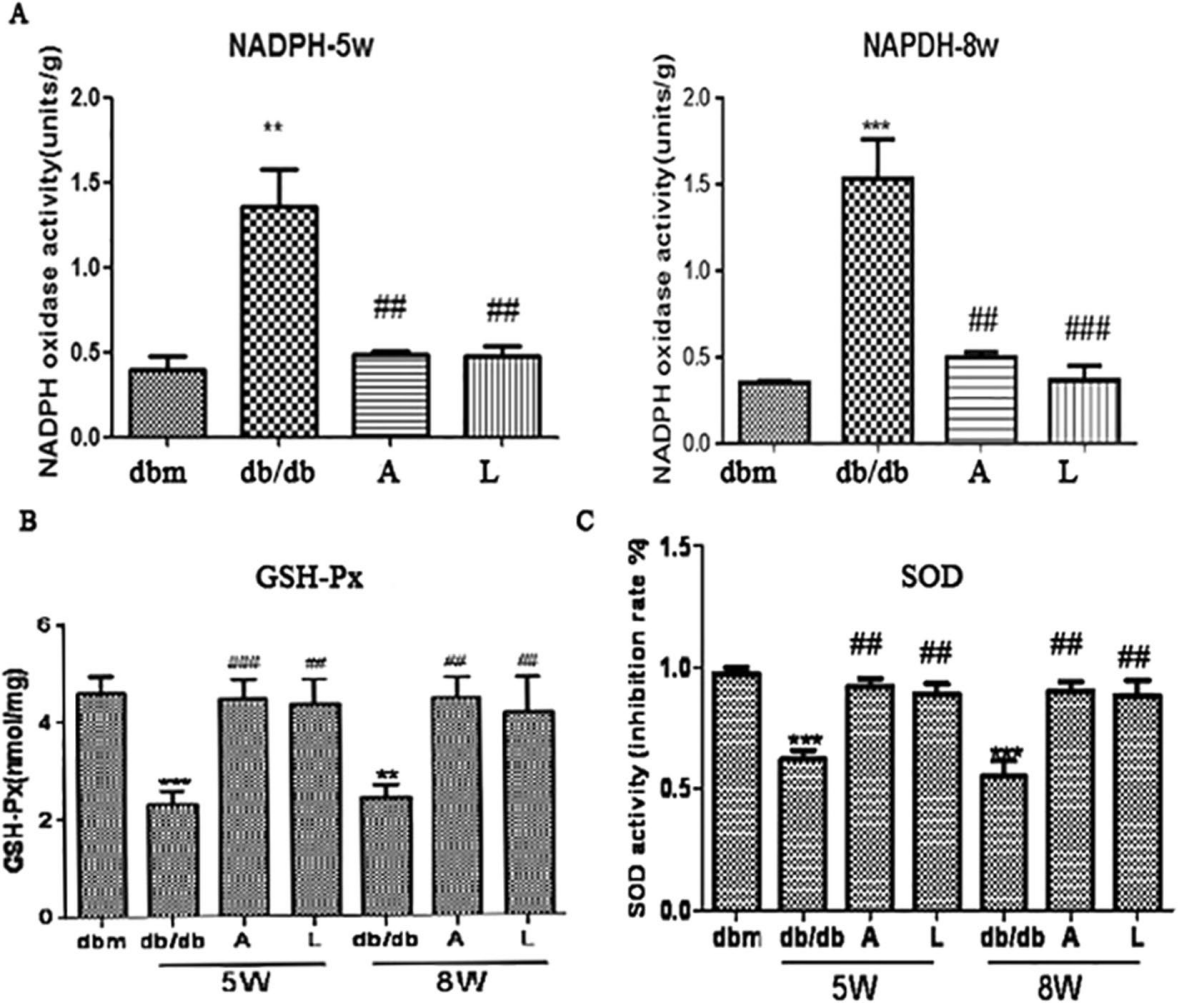
AKF-PD significantly decreases the activity of NADPH oxidase in the kidney of *db*/*db* DN mice. (**A**) Renal NADPH oxidaseactivity was determined in *db*/*m* mice and *db*/*db* mice with the indicated treatments (n = 6 for each group). Means ± SD (standard deviation) are graphed. (**B**,**C**) Renal GSH-Px and SOD activities were measuredin *db*/*m* mice and *db*/*db* mice with the indicated treatments (n = 6 for each group). Means ± SD (standard deviation) are graphed. *p < 0.05, **p < 0.01, ***p < 0.0001 in comparison to *db*/*m* mice; ^#^p < 0.05, ^##^p < 0.01, ^###^p < 0.0001 in comparison to *db*/*db* mice.

OS is a major cause of DN under diabetes conditions^30^. Significant elevations of the renal NAPDH oxidase activity was demonstrated in mock-treated*db*/*db* mice at weeks 5 or 8 compared to *db*/*m* mice (Fig. 2A). Administration of either AKF-PD or losartan to both 5 weeks and 8 weeks *db*/*db* mice significantly reduced renal NAPDH oxidase activity to the basal level detected in *db*/*m*mice (Fig. 2A). ROS is regulated by cell’s activities in ROS production and clearance, suggesting that AKF-PD could also affect ROS elimination. Consistent with this possibility, the activity of renal glutathione peroxidase (GSH-Px) was dramatically increased in *db*/*db* mice when animals were received either AKF-PD or losartanat at weeks 5 and 8 (Fig. 2B). Similar observations were also obtained for superoxide dismutase (SOD) activity (Fig. 2C). Collectively, the above observations suggest the notion that AKF-PD offer a better antioxidation effects during diabetes nephropathy progression.

**Figure 3:**
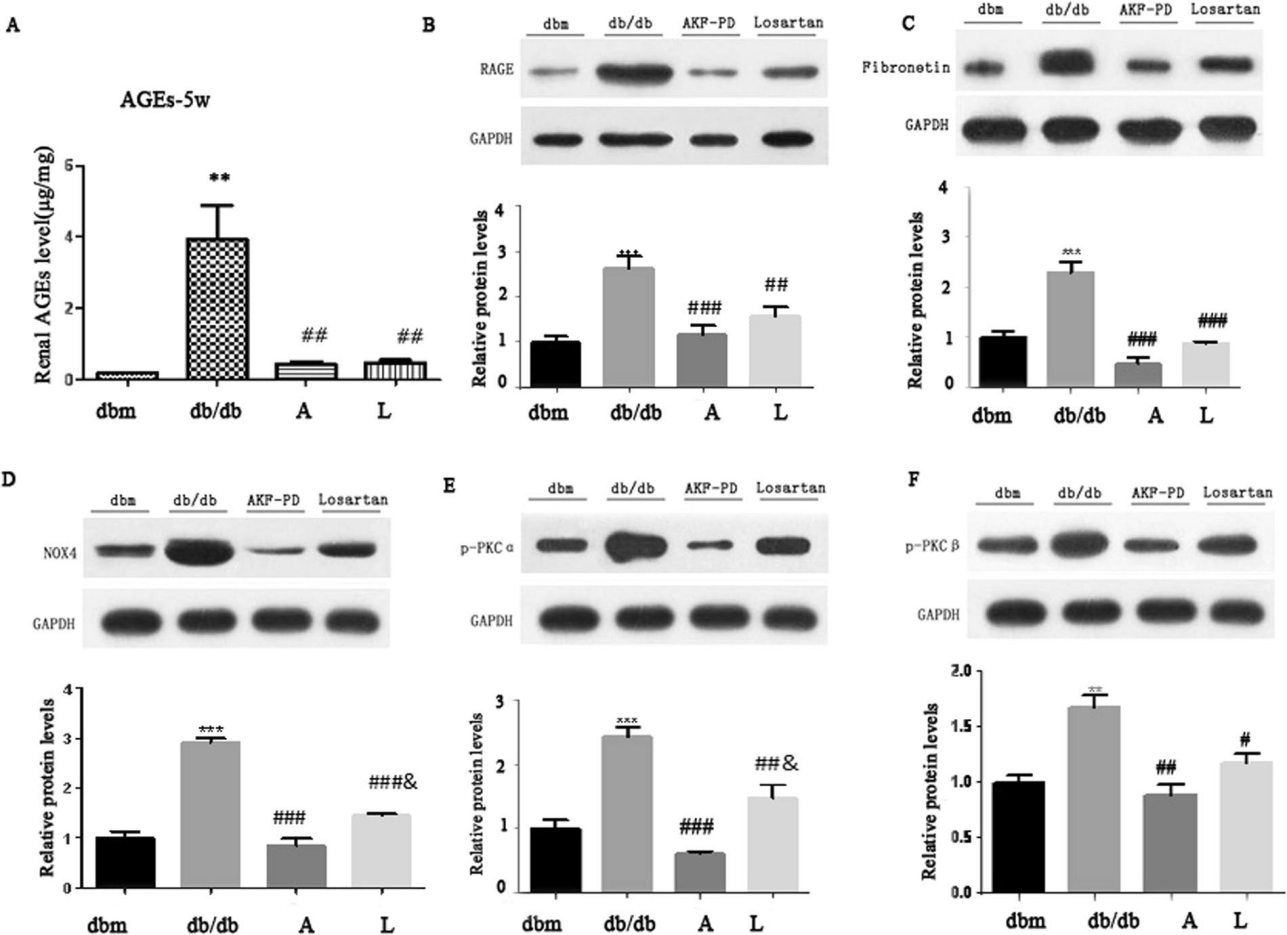
The intervention of AKF-PD initiated at 5-weeks reduces activation of the AGE and *PKC* pathways. Mice with the indicated genotypes were treated as indicated (n = 6 per group). Renal AGEs (**A**), RAGE (**B**), Fibronectin (**C**), NOX4 (**D**), PKCα, and PKCβ expressions were determined; typical images are provided (**B**–**F**); the expression of individual proteins is normalized to specific GAPDH and graphed as relative alterations to *db*/*m* mice (**B**–**F**). *p < 0.05, **p < 0.01, ***p < 0.0001 in comparison to *db*/*m* mice; ^#^p < 0.05, ^##^p < 0.01, ^###^p < 0.0001 in comparison to *db*/*db* mice.

**Figure 4:**
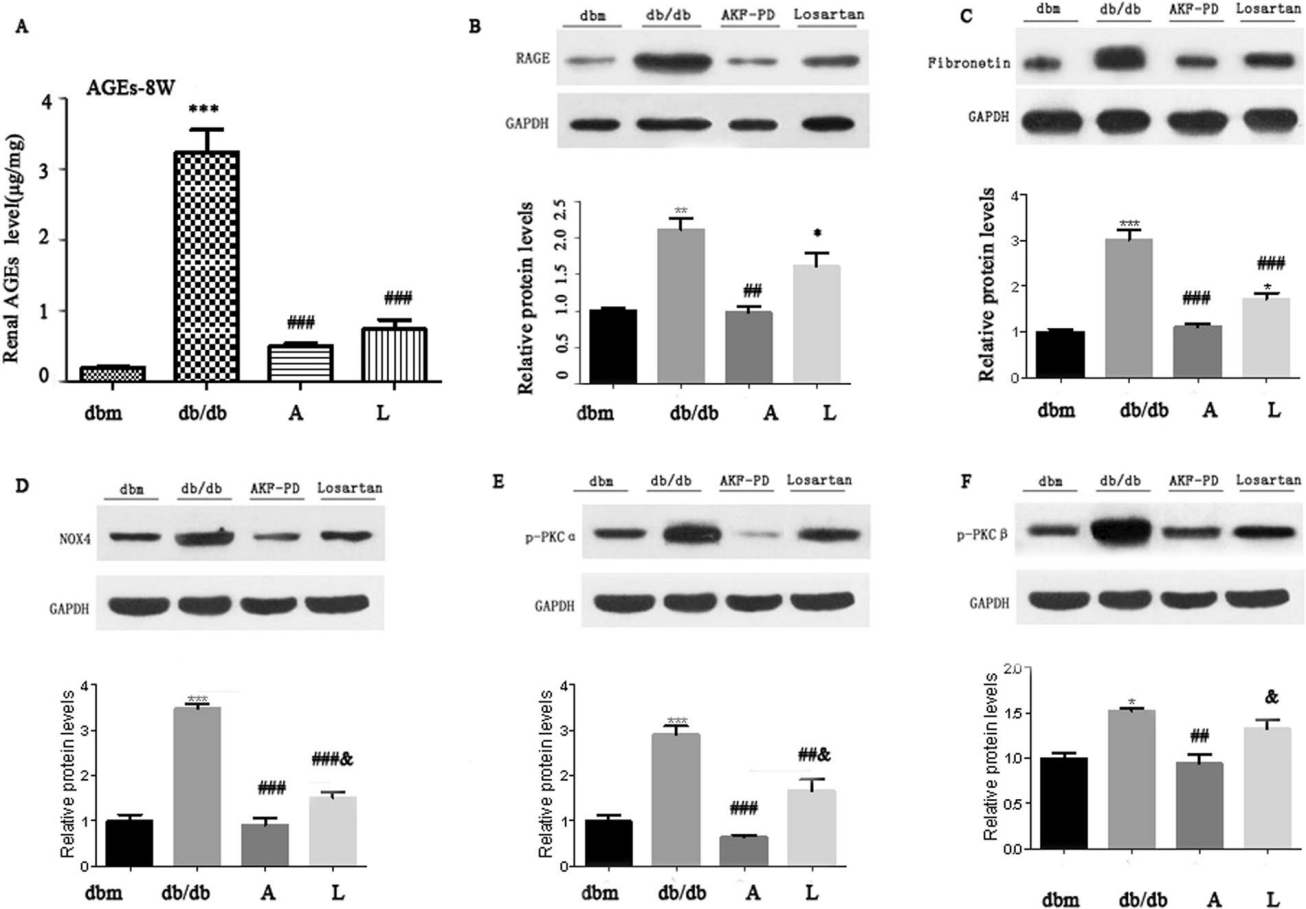
The intervention of AKF-PD initiated at 8-weeks attenuates activation of the AGE and *PKC* pathways. Images are prepared as described in Fig. 3 legend for the indicated treatments started at 8-weeks old animals. *p < 0.05, **p < 0.01, ***p < 0.0001 in comparison to *db*/*m* mice; ^&^P < 0.05, in comparison to Losartan group. ^#^p < 0.05, ^##^p < 0.01, ^###^p < 0.0001 in comparison to *db*/*db* mice. ^&^P < 0.05, ^##&^P < 0.01, ^###&^P < 0.0001 in comparison to db/db mice.

Oxidative stress is plays a pivotal role in the progression of diabetes renal injury. OS is functionally related to advanced glycation end products (AGEs), their receptor RAGE, fibronetin, PKCα, and PKCβ^17^. To further investigate the impact of AKF-PD on renal OS, we hypothesized that AKF-PD driven blockage of oxidative stress in db/db mice was likely mediated by antagonizing AGEs/RAGE and PKC signaling. We detected a dramatic upreglaton of AGEs, RAGE, fibronectin, PKCa, and PKCβ in the kidneys of *db*/*db* mice compared to *db*/*m* mice; all these increases were dramatically reduced to the basal levels observed in *db*/*m* when AKF-PD was given to either 5 weeks or 8 weeks *db*/*db* mice (Figs 3 and 4). These results are even more significant when they are interpreted in the context of experimental duration of 24 weeks, i.e. starting of the AKF-PD intervention at 8 weeks at time when renal damage was clear (Fig. 1) and kidney functions were substantially compromised (Table 1) was sufficient to completely prevent these OS-associated events (Figs 3 and 4). In this regard, we noticed that AKF-PD was superior to losartan in reduction of all the aforementioned events (Figs 3 and 4). Collectively, the results demonstrated a robust potential of AKF-PD in inhibiting renal OS during diabetes progression.

### AKF-PDreduces activation of the AGE-RAGE and PKC pathway

**Figure 5:**
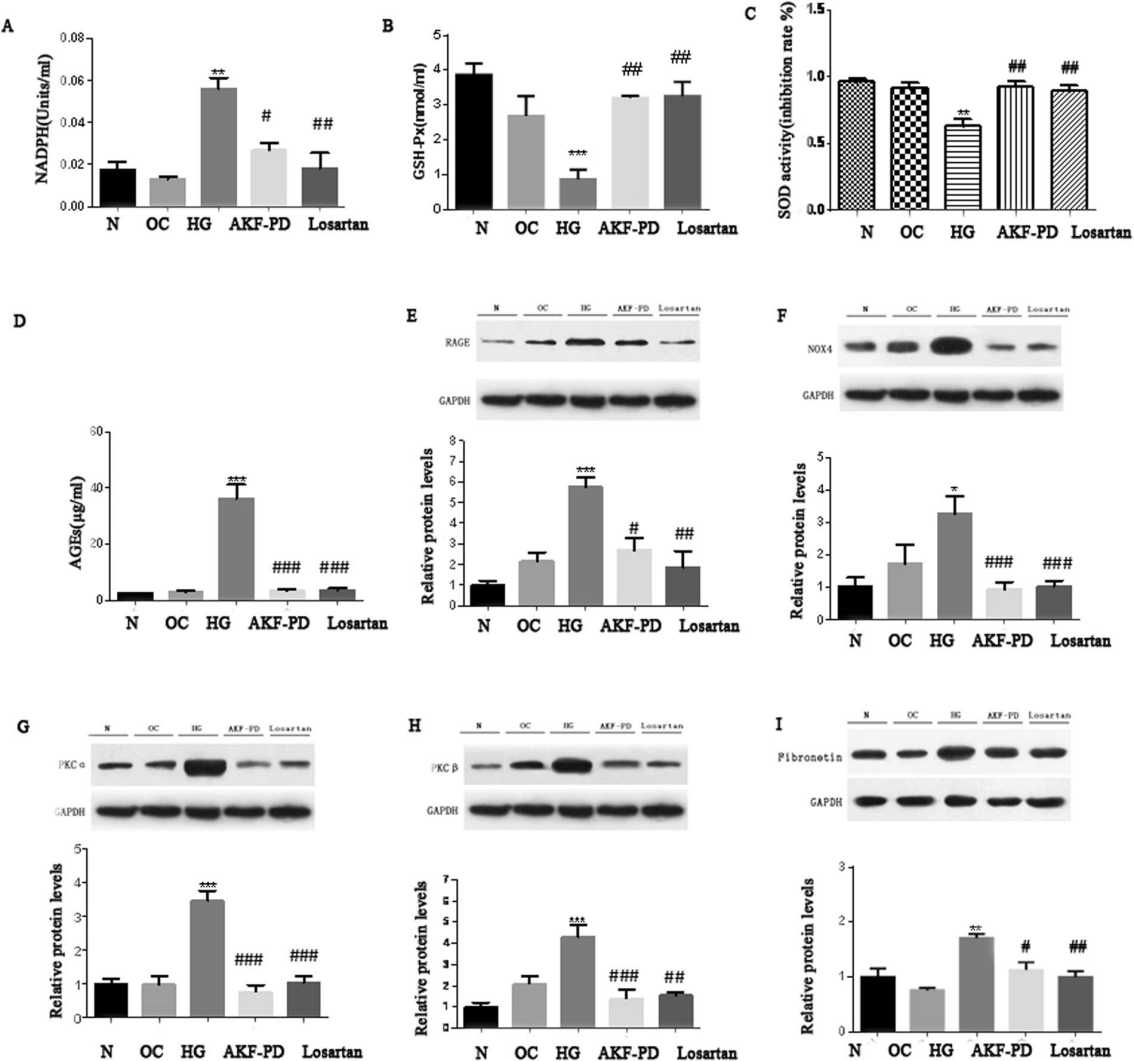
AKF-PD reduces OS-related alterations in HMCs treated with high glucose (HG). Human mesangial cells (HMCs) are treated with 5.6 mM glucose (N), 5.6 mM glucose plus 19.4 mM D-mannitol (OC), 25 mM glucose (HG), HG + AKF-PD (AKF-PD), or HG + Losartan (Losartan) for 24 h. (**A**–**C**) Activities for the indicated enzymes were measured and quantified. Means ± SD are graphed. (**D**–**I**) The indicated proteins were determined by Western blot, normalized to GAPDH, and graphed. Experiments were repeated three times; means ± SD are graphed. *p < 0.05, **p < 0.01, ***p < 0.0001 in comparison to *db*/*m* mice; ^#^p < 0.05, ^##^p < 0.01, ^###^p < 0.0001 in comparison to *db*/*db* mice.

The AGE-RAGE axis and the PKC pathway contribute to OS in part through activation of NAPDH We observed a robust inhibition of AGEs, RAGE, NAPDH Oxidase 4, PKCα, and PKCβ upregulations in the kidney of AKF-PD treated *db*/*db* mice (Figs 2–4). To demonstrate a direct role of AKF-PD in inhibition of these proteins, we showed that high glucose (HG), a hallmark of diabetes, significantly increased NAPDH activity, and reduced GSH-Px and SOD activities in in human renal mesangial cells (HMCs), indicative of OS (Fig. 5A–C). AKF-PD significantly reduced these protein expression (Fig. 5A–C). Furthermore, HG upregulated AGEs, RAGE, PKCα and PKCβ along with an elevation of NOX4 expression in HMCs (Fig. 5D–G); AKF-PD reversed all these alterations (Fig. 5D–G). A major outcome of renal OS under diabetes is to cause ECM expansion. Of note, upregulation of renal fibronectin occurred in *db*/*db* mice and AKF-PD reduced fibronectin accumulation (Figs 3 and 4). Consistent with these *in vivo* observations, HG induced fibronectin expression in HMCs, which was reduced by AKF-PD (Fig. 5I). Collectively, these observations largely supported the results obtained *in vivo* (Figs 2–4), supporting the possibility that AKF-PD reduces renal OS induced by diabetes at least in part via attenuation of the AGE-RAGE-NOX and PKC-NOX pathways.

**Figure 6:**
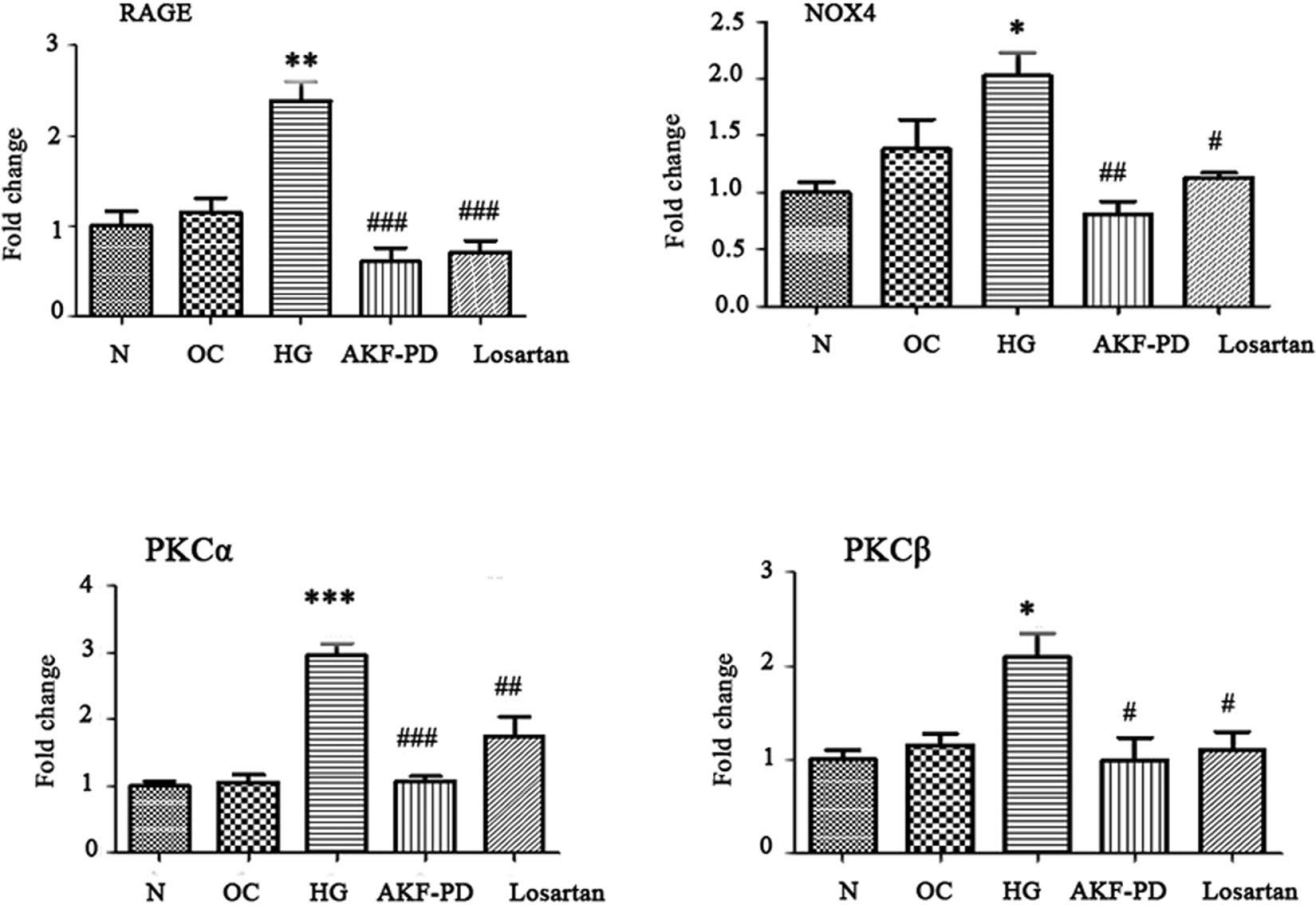
AKF-PD downregulates the expression of ROS related genes in human mesangial cells. HMCs were treated as indicated, followed by real-PCR analysis for the indicated gene expression. Experiments were performed as triplicates and repeated three times. ^*^p < 0.05, ^**^p < 0.01, ^***^p < 0.0001 in comparison to *db*/*m* mice; ^#^p < 0.05, ^##^p < 0.01, ^###^p < 0.0001 in comparison to *db*/*db* mice.

To determine the mechanisms underlying AKF-PD-derived inhibition of the AGE-RAGE-NOX and PKC-NOX pathways, we demonstrated that AKF-PD reduced RAGE, NOX4, PKCα, and PKCβ mRNA expression in HMCs treated with HG (Fig. 6), suggesting that AKF-PD downregulates the AGE-RAGE-NOX and PKC-NOX pathways in part via inhibition of transcription.

### AKF-PD confers protection on mitochondrial damage

**Figure 7:**
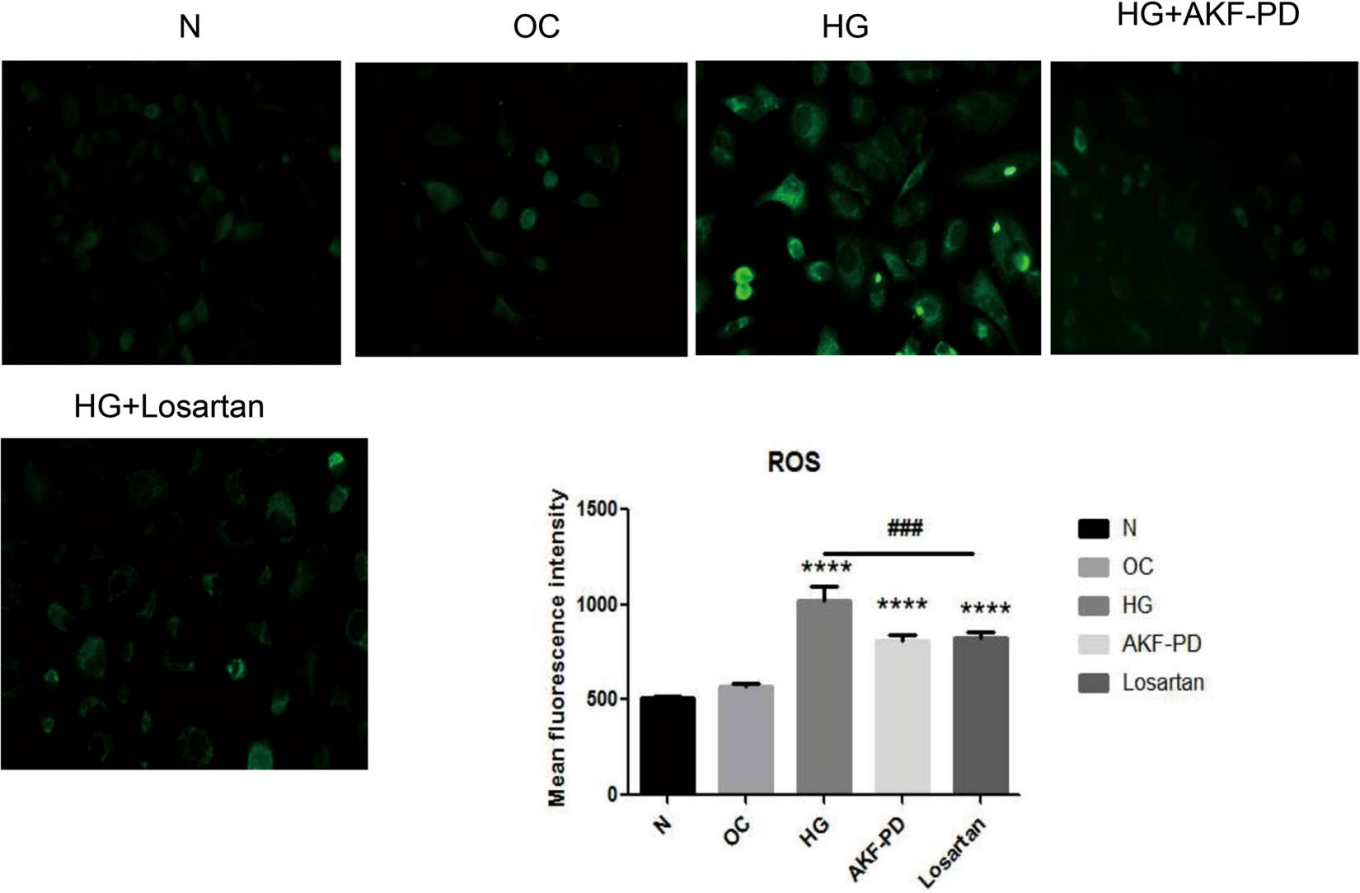
AKF-PD reduces ROS in HG-treated HMCs. HMCs were treated as indicated. Cellular ROS was probed by Dichlorodihydrofluorescein diacetate assay which producedgreen fluorescence directly proportional to the intracellular ROS production. *p < 0.05, **p < 0.01, ***p < 0.000 in comparison to *Ctrl group*; ^#^p < 0.05, ^##^p < 0.01, ^###^p < 0.0001 in comparison to HG-treated group.

**Figure 8:**
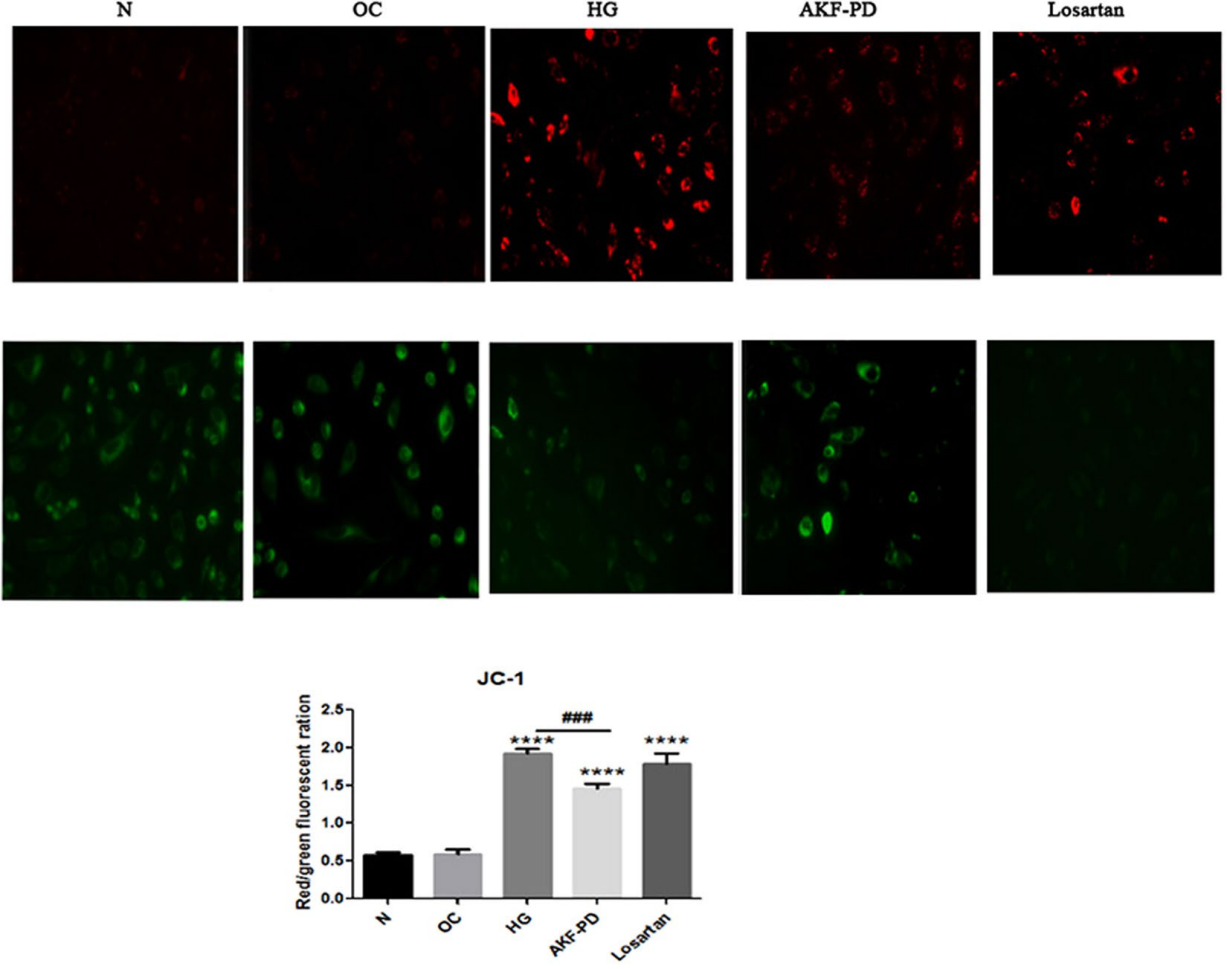
AKF-PD maintains mitochondrial integrity in HMCs treated with HG. HMCs were treated as indicated, followed by measuring mitochondrial potential using the JC-1 assay (see Materials and Methods for details). Elevation of mitochondrial potential (indicative of mitochondrial damage) is indicated by the ratio of red/green. *p < 0.05, **p < 0.01, ***p < 0.000 in comparison to *Ctrl group*; ^#^p < 0.05, ^##^p < 0.01, ^###^p < 0.0001 in comparison to *HG-treated group*.

In line with the above observations, HG resulted in an increase in cellular ROS in HMCs and AKF-PD reduced ROS accumulation (Fig. 7). Mitochondria is the central source of ROS, implying a role of AKF-PD in protecting mitochondrial integrity. We observed that in HG-induced HMCs caused an elevation of ROS along with an increase in mitochondrial potential, indicative of mitochondrial damage (Fig. 8). AKF-PD reduced ROS production and mitochondiral potential in HMCs treated with HG (Fig. 8); this suggests that AKF-PD attenuates ROS production in response to HG environment in part via maintaining mitochondrial integrity. On the other hand, while losartan was able to reduce ROS in HG-treated HMCs (Fig. 7), it had no effects on mitochondrial damage (Fig. 8), supporting AKF-PD being superior to losartanin protecting renal OS.

### AKF-PD preserves mitochondrial integrity via complex mechanisms

**Figure 9:**
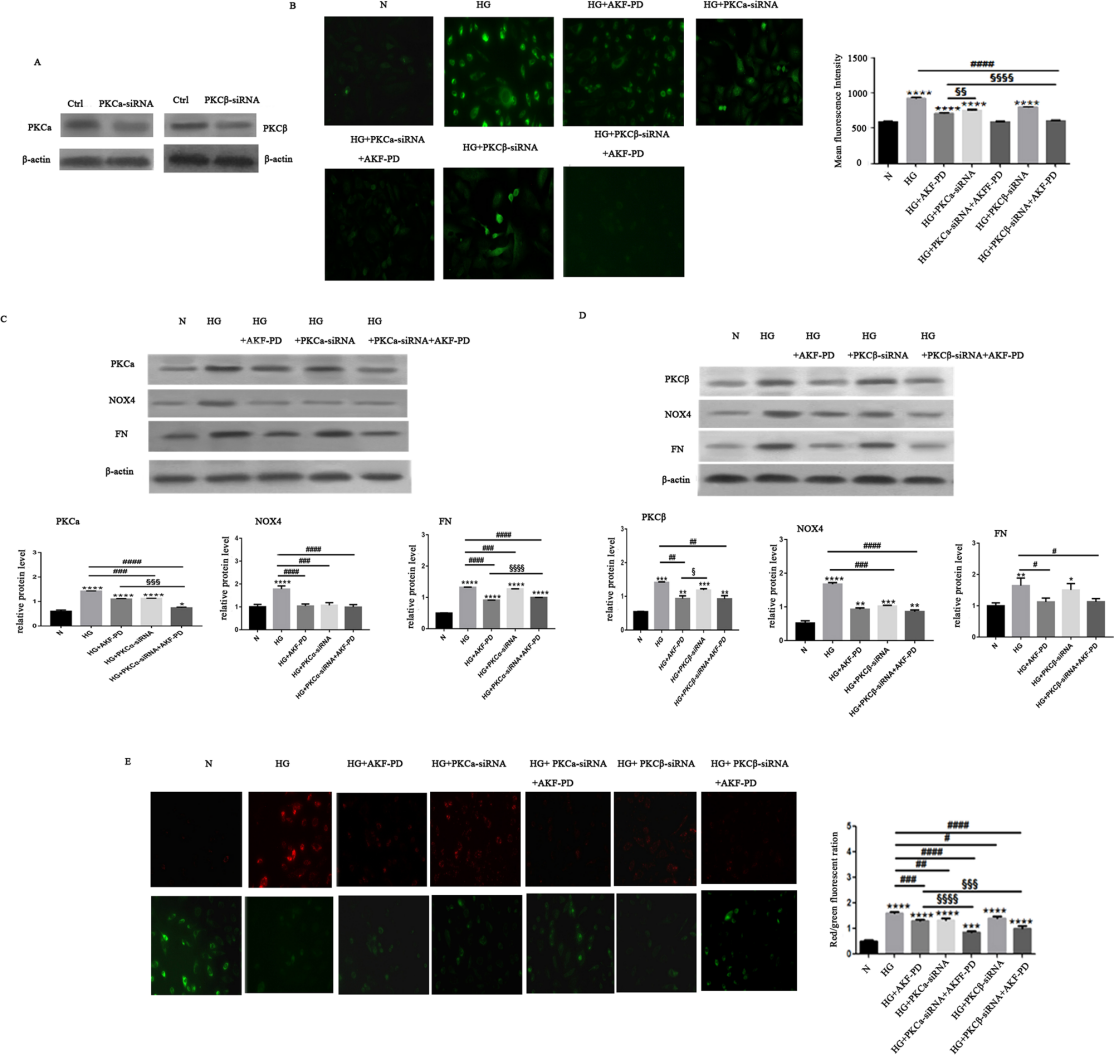
AKF-PD prevents mitochondrial damage in HG-treated HMCs through PKC-dependent and –independent mechanisms. (**A**) HMCs were treated with control (Ctrl) siRNA, PKCα-siRNA, using transient transfection. (**B**,**C**) The intended knockdown was demonstrated. Ctrl-siRNA, PKCα-siRNA HMCs were treated as indicated, followed by measuring ROS with Dichlorodihydrofluorescein diacetate assay, NOX4 and Fibronectin expressions were determined. (**A**) HMCs were treated with control (Ctrl) siRNA, PKCβ-siRNA, using transient transfection. (**D**) The intended knockdown was demonstrated. Ctrl-siRNA, PKCβ-siRNA, or PKCβ-siRNA HMCs were treated as indicated, followed by measuring ROS with Dichlorodihydrofluorescein diacetate assay, NOX4 and Fibronectin expressions were determined. (**E**) Ctrl-siRNA, PKCα-siRNA, or PKCβ-siRNA HMCs were treated as indicated, followed by measuring ROS with Dichlorodihydrofluorescein diacetate assay. Typical images were acquired using a fluorescence microscope. Experiments were repeated three times, mean fluorescence intensities ± SD are graphed. (**C**) Ctrl-siRNA, PKCα-siRNA, or PKCβ-siRNA HMCs were treated as indicated. Mitochondrial potential was determined. Experiments were repeated three times; mitochondrial potentials are graphed.

Our observed concurrent occurrence between PKC (alpha and beta) upregulations and NOX4 increases *in vivo* (Figs 3 and 4) and *in vitro* (Figs 5 and 6) suggests that the PKC pathway is involved in AKF-PD-mediated protection of mitochondrial integrity. To examine these potential mechanisms, we have individually knocked down PKCα and PKCβ (Fig. 9A). Knockdown of either significantly reduced ROS levels, fibronectin (FN), NOX4 in HG-treated HMCs (Fig. 9B–D), demonstrating critical roles of PKCα and PKCβ in ROS production in HMCs under HG conditions. These results are consistent with the demonstrated importance of PKCα and PKCβ in ROS production^3^. While AKF-PD clearly reduced ROS in HMCs treated with HG, the ROS level in HMCs treated with both HG and AKF-PD was marginally lower than HG-induced ROS level and NOX4 detected in HMCs with knockdown of either PKCα or PKCβ (Fig. 9B, comparing the HG + AKF-PD bar with the HG + PKCα-siRNA bar or the HG + PKCβ-siRNA bar). Interestingly, the combination of AKF-PD with knockdown of either PKCα or PKCβ further reduced ROS in HG-stimulated HMCs to the basal level (Fig. 9B).

We subsequently determined the contributions of PKCα and PKCβ to AKF-PD-derived protection of mitochondrial damage. The similar relationship among AKF-PD and knockdown of either PKCα of PKCβ was observed with respect to mitochondrial potential (Fig. 9E) and ROS levels (Fig. 9B) in HMCs in the presence of HG. Nonetheless, the combination of AKF-PD with knockdown of either PKCα or PKCβ partially prevented increases of mitochondrial potential (Fig. 9E). Collectively, the above results suggest that AKF-PD inhibits ROS production and protects mitochondria from damage in HG-treated HMCs through PKCa-dependent and PKCβ–independent mechanisms.

## Discussion

Our research provides the first thorough evidence for AKF-PD being such a drug. AKF-PD is a pyridyl ketone compound with a broad spectrum of antifibrosis activity^3,31^. Previous research has revealed its utility in inhibition of mouse renal fibrosis caused by diabetes and unilateral ureter obstruction^3,13^. In this study, we observed that AKF-PD coordinately reduces renal OS in *db*/*db* mice through preventing upregulations of the NADPH oxidase and downregulations of the anti-oxidative enzyme system like GSH-Px and SOD. Considering ROS promoting DN development through four recognized pathways: AGEs, PKCs, Polyalcohol, Hexosamine pathways^30,32^, we demonstrated that AKF-PD inactivates the pathways of AGE and PKC, supporting AKF-PD possessing potent activities in inhibiting renal OS. However, we cannot exclude the possibility that AKF-PD also antagonizes the other two pathways; this possibility deserves investigations in the future.

In patients with diabetes, high glucose enhances the generation of ROS in mitochondria, induces OS within histiocyte, and ultimately promotes the development of various complications. ROS is largely originated from mitochondria as O^−^, which can inhibit the activity of the enzyme complex II, II, and III in the electron transport chain and then lead to energy synthesis dysfunction. In this process, the mitochondria inner membrane is vulnerable, probably due to its special biological and anatomic characteristic^33^. Our previous studies have verified that AKF-PD could depress the production of ROS and suppress the expression of lipid peroxidation product (MDA and 8-iso-PGF2a), which raises the hypothesis that AKF-PD attenuates DN through alleviating the renal mitochondrial oxidative injury^27^. This hypothesis is supported by our results that AKF-PD almost normalized JC-1 aggregation in the membrane of mitochondrialin HMCs while Losartan partly reversedmitochondrial damage induced by HG.

The mechanisms underlying AKF-PD-derived protection and therapeutic value towards DN need further investigation. Nonetheless, our research suggests these mechanisms are PKCα and PKCβ-dependent and PKCα, PKCβ-independent. AKF-PD was able to HG-caused mitochondrial damage and ROS levels in HMCs with knockdown of either PKCα or PKCβ. These observations supports the contributions of pathways independent of either PKCα or PKCβ to AKF-PD activities in maintaining mitochondria integrity; alternatively PKCα or PKCβ is able to compensate the knockdown of another.

In conclusion, we demonstrate that AKF-PD displays an impressive preservation of renal function in 24 weeks old *db*/*db* mice when intervention was started from the age of 5 weeks or 8 weeks. In view ofthe further reduction of ROS levels and protection of mitochondrial potential alterations when AKF-PD was combined with knockdown of PKCα or PKCβ, the clinical potential of this combination could be explored in the future.

## Materials and Methods

### Mouse model

Four-week old male *db*/*db* mice (n = 36) were fed and randomly divided into six groups. Mice in the AKF-PD groups were treated with 500 mg/kg/day AKF-PD at the age of 5 weeks (AKF-PD-5w group) or 8 weeks (AKF-PD-8w group); the dosage was chosen according to on our previously defined conditions^34,35^. Mice in the positive control groupswere treated with 20 mg/kg/d losartan at the age of 5 weeks (LOS-5w group) or 8 weeks (LOS-8w group). Mice in the negative control groups were treated with placebo at the age of 5 weeks (Ctrl-5w group) or 8 weeks (Ctrl-8w group). Mice with the *db*/*m* genotype (db/m group, n = 6) served as untreated blank control. All treatments were performed daily by oral gavage. After the treatment ended, mice were sacrificed at 24 weeks old (Fig. 1A), and kidney tissues were collected for pathological, protein and mRNA detection. The protocol was approved by the Committee on the Ethics of Animal Experimentation and Care of the Central South University Xiangya Hospital.

### Evaluation of Renal Function

Blood total triglyceride (TC), total cholesterol (TG), glucose (GLU), glycosylated hemoglobin (GSP), serum creatinine (SCR), blood urea nitrogen (BUN) and blood uric acid (BUA) as well as 24-hour albumin/creatinine ratio (ACR) were measured every 2 week. Body weight (BW) was monitored every day.

### Pathology and immunohistochemistry

Histological Evaluation were performed as previously described study^36,37^. Hematoxylin-eosin (HE) and Periodic Acid-Schiff (PAS) staining were carried out for pathological analysis. Mesangial expansion was determined by glomerular matrix expansion index (GMI) which was calculated as our previous studies^3^. The expression of FN in kidney tissue was detected by western blot analysis.

### Cell culture

Human renal mesangial cells (HMC) were provided by Changsha Central Hospital laboratory. HMCs cells were cultured in DMEM supplemented with 8% FBS, penicillin (100 U/ml) and 100 μg/ml streptomycin (Invitrogen), at 37 °C in a humidified atmosphere of 5% CO2 and 95% air. The cells were seeded on six-well culture plates to 60–70% confluence in complete medium containing 5% FBS for 24 h. Cells were divided into 5 groups to receive different drug interventions respectively: N group treated with 5.6 mM glucose, OC (mannitol) group treated with 5.6 mM glucose and 19.4 m MD-mannitol, HG group treated with 25 mM glucose, AKF-PD group treated with 25 mm glucose and 2 mM AKF-PD, and losartan group treated with 25 mM glucose and 2 μM losartan.

### RNA extraction and real-time RT-PCR

RNA was isolated from sample using Trizol (Invitrogen, Carlsbad, CA, USA) according to previous publications^38^. Total RNA was purified with RNAesy Micro columns (Qiagen, Valencia, CA, USA), and was reverse-transcribed using the High-Capacity cDNA reverse transcription kit (Taraka, Dalian, China). Quantitative real-time PCR was performed using SYBR Green I (Taraka, Dalian, China) (Applied Biosystems Step-one TM Real-Time PCR System) in triplicates and quantified using the ΔΔCt method. 500 ng cDNA per reaction was used, and the expressions data of RAGE, NOX4, PKCα, PKCβ, and fibronectin (FN) mRNA were normalized to GAPDH expression as the internal control. The melting temperature analysis and linear amplification with increasing PCR cycles were done for the validity of amplification.

### Western blot analysis

Total protein extracts from kidney tissue were prepared using the Protein Kit (Qiagen, Valencia, CA, USA) according to manufacturer’s instructions and previous publications^39,40^. Tissues were processed in liquid nitrogen and lysed with SDS-PAGE sample buffer. To detect the protein expression of AGEs, RAGE, p-PKCα, p-PKCβ, NADPH-NOX4, Fibronetin, 30 μg protein lysates were loaded per well, separated on 8% or 12% SDS-polyacrylamide gels and transferred to PVDF membranes (Immobilon P, Millipore, Bedford, MA). The membranes were blocked with 5% fat free milk in Tris-buffered saline containing 0.1% Tween-20 (TBST) for 30 minutes at room temperature, and then probed with antibodies, followed by incubation with horseradish peroxidase-conjugated secondary antibodies (1:6000) at room temperature for 1 hour. The immune complexes were visualized by chemiluminescence using an ECL kit (Pierce, USA). Positive bands were analyzed with Quantity One software (Bio-Rad). GAPDH was used as the internal control, and all the experiments were repeated for three times.

### OS measurement

Kidney tissue suspension was extracted and cytochrome C reduction method was used to measure the NAPDH oxidase activity. SOD and GSH-Px activity was detected by NBT method and DNTB method, respectively. The metabolic product peroxidation were examined by western blot analysis. The measurement of intracellular ROS level changes we reperformed as previously described^13^. Each experiment was performed in triplicate.

### Detection of mitochondrial function

Mitochondrial membrane potential was determined using the fluorescent probes JC-1.JC-1 could selectively enter mitochondria and reversibly changes color as membrane potentials increase (range 80–100 mV) which result from the reversible formation of JC-1 aggregates upon membrane polarization. This progress would cause shifts in emitted light from 530 nm (i.e., emission of JC-1 monomeric form) to 590 nm (i.e., emission of J-aggregate) when excited at 488 nm. Both colors can be detected using filters for FITC and PE/phycoerythrin/rhodamine, respectively.

### Statistical analysis

All the data analysis was conducted using SPSS 17.0 software (SPSS Inc, Chicago, IL, the USA). Continuous and categorical data was presented as umber (percent) or mean ± *SD (*standard derivation*)*. Normal distribution test and homogeneity test of variances should be conducted for the data of different groups at first. If data meet the normal distribution (P > 0.20) and homogeneity of variance (P > 0.10), one-way analysis of variance (ANOVA) assay was used to compare difference groups. If not, the rank sum test should be conducted. The P-values of less than 0.05 were considered to be statistically significant.
